# Adenovirus 36 and Obesity: An Overview

**DOI:** 10.3390/v7072787

**Published:** 2015-07-08

**Authors:** Eleonora Ponterio, Lucio Gnessi

**Affiliations:** Department of Experimental Medicine, Section of Medical Pathophysiology Food Science and Endocrinology, Sapienza University of Rome, 00161 Rome, Italy; E-Mail: lucio.gnessi@uniroma1.it

**Keywords:** Adv36, obesity, human virus, immune response

## Abstract

There is an epidemic of obesity starting about 1980 in both developed and undeveloped countries definitely associated with multiple etiologies. About 670 million people worldwide are obese. The incidence of obesity has increased in all age groups, including children. Obesity causes numerous diseases and the interaction between genetic, metabolic, social, cultural and environmental factors are possible cofactors for the development of obesity. Evidence emerging over the last 20 years supports the hypothesis that viral infections may be associated with obesity in animals and humans. The most widely studied infectious agent possibly linked to obesity is adenovirus 36 (Adv36). Adv36 causes obesity in animals. In humans, Adv36 associates with obesity both in adults and children and the prevalence of Adv36 increases in relation to the body mass index. *In vivo* and *in vitro* studies have shown that the viral E4orf1 protein (early region 4 open reading frame 1, Adv) mediates the Adv36 effect including its adipogenic potential. The Adv36 infection should therefore be considered as a possible risk factor for obesity and could be a potential new therapeutic target in addition to an original way to understand the worldwide rise of the epidemic of obesity. Here, the data indicating a possible link between viral infection and obesity with a particular emphasis to the Adv36 will be reviewed.

## 1. Introduction

Obesity is a disease characterized by an excess of fat mass. The incidence of obesity in recent decades has grown dramatically [1].

The prevalence of obesity doubled in adults and tripled in children during the last 20 years, even in countries with traditionally low rate of the disease. In 2014, more than 1.9 billion adults, 18 years old and older, were overweight. Of these, over 600 million were obese. The worldwide prevalence of obesity more than doubled between 1980 and 2014. (World Health Organization (WHO) data; [2]). The risks of diabetes, hypertension and dyslipidaemia increase starting from a body mass index (BMI) of about 21.0 kg/m^2^, thereby reducing life expectancy and greatly increasing the health and social economic burden. Obesity is clearly related to increased mortality, morbidity and disability rates. Overweight and obesity are the fifth leading risk for global deaths. Obesity is now described as a phenomenon in character epidemic [3], and according to the WHO, obesity represents “one of the major Public Health problems of our time”. Obesity is also a risk factor for numerous chronic diseases such as cardiovascular diseases and diabetes, orthopedic problems and mental disorders. The scientific community started to study the obesity epidemic looking at the possible causes with the aim to find effective treatments to reduce morbidity and mortality.

Although obesity is a multifactorial disease caused by the interaction between genetics, metabolism, social, cultural and environmental factors, viral infection has been suggested as a possible cofactor for the development of obesity [4].

Immune response depends by nutritional status and can be easily dysregulated in states of imbalanced nutrition such as obesity. This may predispose obese individuals to increased susceptibility to infection [5,6].

In the hospital setting, obese patients are more likely to develop secondary infections and complications such as sepsis, pneumonia, bacteremia, wound infections, and respiratory infections [7,8]. Obesity has been associated with increased risk of complications due to surgical site infections [9,10,11]. Obese individuals have increased risk of *Helicobacter pylori* [12] infection and overweight children show a three times greater risk of *Neisseria meningitides* [13]. Obesity is also a risk factor of severe infection and death caused by the pandemic influenza strain H1N1 [14].

Overall, these observations indicate that excessive adipose tissue expansion predisposes individuals to various infections. On the other hand, new data have been generated in the last few years suggesting infectious agents being the cause of obesity in addition to being more easily hosted in an obese individual. Among the multitude of infectious agents, adenoviruses are the human pathogens that more than others are causatively and correlatively linked with animal and human obesity, respectively, and seem to directly influence the adipose tissue [15,16].

In animals, adenovirus serotype Adv31 and Adv9 correlate with obesity and are adipogenic in animal cells culture [17,18]. An avian adenovirus (SMAM-1) and the human adenovirus type 36 (Adv36) have been associated with obesity [19] and there are reports a suggesting significant role of adenoviruses in the development of human obesity.

Here we provide an overview of the data available on the relationship between adenovirus infection and obesity.

## 2. Association of Viral Infections with Obesity in Animal Models

Five infectious agents were implicated in contributing to obesity, including canine distemper virus (CDV), rous associated virus (RAV)-7, borna disease virus (BDV) and adenoviruses [19]. Apart from adenoviruses, all the other viruses of this list are associated with brain damage and, in experimental animals, obesity develops via brain involvement or via direct damage of fat tissue. CDV is a paramyxovirus that infects dogs and other wild mammals; it was one of the first infectious agents identified to cause enlarged fat cells and increased body weight in mice [19]. The CDV infected animals showed twofold increase in body weight and a selective, virus-induced disruption of critical brain catecholamine pathways [20]. RAV-7 is one of the most common retroviruses affecting chickens [21]. The infected chickens are smaller than hatchmates and develop obesity, ataxia, lymphoblastoid infiltration of thyroid and pancreas, liver fat accumulation and a frank lipemia. BDV is a nonsegmented negative-stranded RNA virus that may cause obesity in various animals, including rats and chickens. Most studies suggest that BDV causes obesity by inflammation of the hypothalamus. In infected animals, hyperplasia of pancreatic islets, increase in glucose and triglyceride levels is also seen [19].

Adenoviruses are the only infective agents reported to be linked with adiposity in both experimental animal models and naturally infected humans [22].

## 3. Adenoviruses

**Figure 1:**
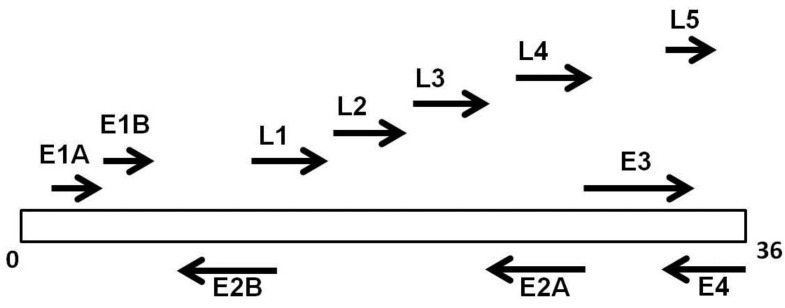
Genomic organization of Adv36: The early proteins (E1A, E1B, E2A, E2B, E3, and E4) are involved in the regulation of replication of DNA. The late proteins (L1–L5), products of the translation of late mRNA, constitute structural capsid proteins.

Adenoviruses are medium size viruses. All human adenoviral genomes have the same general organization. The genome consists of a single linear, double-stranded DNA molecule with short inverted terminal repeats at each end. A protein, terminal protein, is covalently attached to both the 5′ ends of the genomic DNA. The viral chromosome has five early transcription units (E1A, E1B, E2, E3 and E4) (Figure 1).

There are more than 50 immunologically distinct serotypes of adenovirus that can cause infections in humans. Serotypes are operationally defined by the ability of the antibodies induced by infection to neutralize that serotype only, and not other adenovirus. The human adenoviruses have been further classified into six subgroups (A–F) based upon hemoagglutination proprieties and genetic relatedness.

Although the epidemiological characteristics of the adenovirus serotype change, all are transmitted by direct contact, fecal-oral transmission, and sometimes by environmental transmission (contaminated water). Most of the adenovirus cause respiratory diseases, but can also cause gastroenteritis, conjunctivitis and cystitis [19,23].

The adenovirus virion contains a 36 kb genome of double-stranded DNA, which is surrounded by a non-enveloped icosahedral protein capsid. The capsid is composed of 252 capsomers: 240 hexons and 12 pentons at the vertices of the icosahedrons. Each penton consists of a penton base and a penton fiber. The fibers consist of a slender shaft with a globular head. They are involved in the process of attachment of the virus particle to the host cell.

Adv2 and Adv5 have been used for most studies of the adenovirus replication cycle. These viruses can be easily grown in the laboratory by infecting permissive cell lines such as HeLa and A549 cells.

Adv5 and Adv37 increase adiposity in animals and have been indicated as possible contributing factors to the rising problem of obesity [24], whereas human adenoviruses Adv2 and Adv31 are not adipogenic in animals. Among the over 50 human adenoviruses available, Adv36 is the most studied also because of its minimal cross-reactivity with other human adenoviruses [25,26,27]. The exact mechanisms leading to the development of obesity through these viruses are unknown. An accurate understanding of the varied etiological factors of obesity (including infecting agents) may lead to cause-specific treatment and successful management of this disease.

## 4. Adv36 and Obesity

### 4.1. In Vivo Studies

The adipogenic effect of adenoviruses has been studied since 1990, when the adipogenic role of the avian adenovirus SMAM-1 in chickens was observed for the first time [28]. SMAM-1 is antigenically similar to chicken embryo lethal orphan virus (CELO), which is common among poultry in the USA. SMAM-1 increases adiposity in experimentally infected chickens and their naive cage-mates, suggesting horizontal transmission of obesity induced by the virus [28]. Paradoxically, SMAM-1-induced adiposity in animals is associated with lower serum cholesterol and triglyceride concentrations compared to uninfected counterparts [29].

The human adenoviruses Adv5 and Adv37 increase the adiposity in animals, while other human adenoviruses like Adv2 and Adv31 are not adipogenic [30,31]. Adv36 is adipogenic in animals [30,32,33,34] and reduces significantly the concentrations of cholesterol and triglycerides compared to uninfected controls. Longitudinal studies in monkeys [33] showed a 15%–30% increase of body weight and a reduction of serum cholesterol after natural infection with Adv36. Adv36 is easily transmitted between animals [35]. After transfusion of a small amount of blood from Adv36 infected chicken to another animal, sharing the same cage, the animals became obese [35].

Pasarica *et al.* [34] showed that rats infected with Adv36 had an increase of weight, insulin sensitivity and glucose uptake. Furthermore, Adv36 infected non-human primates [33], had increase of weight and anti-Adv36 antibodies, but a decrease of total cholesterol. Similar results were obtained in marmosets and a decrease of total cholesterol was found in hamsters [36].

Rats inoculated with Adv36 or UV-inactivated Adv36 [37], showed 23% greater epididymal fat pad and viral mRNA and DNA were detected in liver, brain and adipose tissue. Either intranasal or intra-peritoneal routes of viral inoculation showed similar results. Adv36 stimulates preadipocyte differentiation [38] and CCAAT/enhancer-binding protein beta (C/EBPβ) expression [39]. Pasarica *et al.* [34] found that Adv36 up-regulated (C/EBPβ) the downstream genes CCAAT/enhancer-binding protein alfa (C/EBPα) and glycerol-3-phosphate dehydrogenase (GPDH) in infected rats, suggesting that the target of Adv36 may be C/EBPβ or genes upstream in the pathway. Adipogenesis is accelerated by progression of adipocyte proliferation and differentiation, and ultimately, cellular signaling pathways are affected [38,40]. The expression of early, intermediate, and later genes of differentiation (such as C/EBPβ) increases, followed by expression of C/EBPα and PPARγ and lipid accumulation [41]. Particularly, increases in C/EBPβ and PPARγ genes and RNA expressions related with adipogenesis [42]. These results offer further support to the up-regulation of preadipocyte differentiation as a mechanism for the adipogenic effect of Adv36.

### 4.2. In Vitro Studies

The infection with Adv36 accelerates differentiation and proliferation of the 3T3-L1 human preadipocytes into adipocytes [27,43,44] and increases the concentration of lipid content in fat cells.

*In vitro* experiments on human stem cells derived from primary adipose tissue/stromal cells (hASC) support the hypothesis that natural infection with Adv36 in hASC cell line increases adiposity. The ability to Adv36 to induce adipogenesis in this cell line can provide additional tools to investigate pathways still unknown [44]. Wang *et al.* suggest that the dual effects of Adv36 on lipid metabolism by reducing fatty acid oxidation and increasing *de novo* lipogenesis, result in fat accumulation in muscle cells that may be mediated by promoting cell death-inducing DFFA-like effector c/FSP27 expression [45]. Further research to explore the adipogenic potential of human adenoviruses is warranted.

## 5. Prevalence of Adv36 in Human Obesity

Six human adenoviruses have been studied in relation to obesity [29,30,31,43] and a number of human studies have shown a correlation between antibodies to the Adv36 and obesity (Table 1). Due to the unique amino acid sequences in protruding region of the hexon (loops 1 and 2) of Adv36 [46] there is a low cross-reactivity between Adv36 and other adenovirus in classical serum neutralization assays thus, the information derived from these studies are reliable. Furthermore, the Adv36 genome has a high stability, which is useful for diagnostic purposes and in the development and application of vaccines and therapeutic reagents [47]. Moreover, bioinformatics comparisons with other human adenoviruses identified significant differences, suggesting unique functions of Adv36 possibly linking Adv36 with adipose tissue [40]. In humans, the natural infection with Adv36 is diagnosed by the presence of the viral DNA in adipose tissue [44,48] or by neutralizing antibodies [49].

Analogously to animals, seropositivity to Adv36 is associated with low level of cholesterol and low triglyceride concentrations in serum of obese individuals [30].

**Table 1:** Studies on the association of Adv36 and obesity. BMI, body mass index; TG, triglycerides; TC, total cholesterol; LDL, low density lipoprotein; HDL, high density lipoprotein; BG, blood glucose; NAFLD, Nonalcoholic fatty liver disease; SBP, systolic blood pressure; WC, waist circumstance; NA, not available; SNA, serum neutralization assay; DXA, dual-energy X-ray absorptiometry; VEGF, vascular endothelial growth factor; MCP-1, monocyte chemoattractant protein-1; TNFα, tumor-necrosis-factor-alpha; IL-6, interleukin-6; HERITAGE, HEalth, RIsk factors, exercise Training And Genetics; PBRC, Pennington Biomedical Research Center; MET, Mechanisms of the Metabolic Syndrome in Prepubertal Youth.

First Author	Country	Parameters	BMI	Subjects	Prevalence of Adv36	Method
**Atkinson, 2005 [49]**	USA	Obesity, BMI, TG, TC	BMI ≥ 30	360 obese and 142 non-obese adults	Obese 30% Non-obese 11%	SNA
**Atkinson, 2005 [49]**	USA	BMI, TG, TC	NA	28 sets of twins	Overall 22%	SNA
**Trovato, 2009 [95]**	ITALY	Obesity, BMI, TG, TC, LDL,HDL, SBP	BMI ≥ 30	68 obese and 135non-obese adults	Obese 65% Non-obese 33%	SNA
**Atkinson, 2010 [56]**	South Korea	TC, WC, SBP, BG	NA	83 obese or overweightchildren and one nonobese child	Overall 30%	SNA
**Broderick, 2010 [50]**	USA	Obesity	BMI ≥ 29	146 obese and 147 non-obese adults	Obese 34% Non-obese 39%	SNA
**Gabbert, 2010 [58]**	USA	Obesity, BMI, WC	BMI.95 thpercentile	67 obese and 57 non obese children	Obese 22% Non-obese 7%	SNA
**Na, 2010 [53]**	South Corea	Obesity, BMI, TG, TC, WC,LDL, HDL, SBP, BG	BMI ≥ 30	259 obese and 59 nonobese children	Obese 29% Non-obese 14%	SNA
**Trovato, 2010 [96]**	ITALY	BMI, TG, TC, LDL, HDL, BG	NA	65 NAFLD and 114 non-NAFLD adults	NAFLD 32% Non-NAFLD 46%	SNA
**Krishnapuram, 2011 [97]**	USA	Fasting insulin, Fasting glucose, Insulin sensitivity, HOMA,	NA	(1) HERITAGE Family Study (n 671)	(1) HERITAGE Family Study 13%	SNA
				(2) PBRC Study (n 206)	(2) PBRC Study 18%	
				(3) MET Study (n 45)	(3) MET Study 22%	
				(4) VIVA LA FAMILIA Study (n 585)	(4) VIVA LA FAMILIA Study 7.1%	
**Goossens, 2011 [51]**	Netherlands	Obesity, BMI	NA	136 obese, 281 nonobese, and 92 BMI-unknown adults	5.5% were positive for Adv36 antibodies, No adenoviral DNA	SNA, PCR
**Na, 2012 [98]**	South Korea	Obesity, BMI, TG, TC, WC, HDL, SBP, BG	BMI ≥ 25	180 obese and 360 non-obese adults	Obese 30% Non-obese36%	
**Trovato, 2012 [99]**	ITALY	BMI, TG, TC, LDL, HDL, BG	NA	62 NAFLD adults	Overall 40%	SNA
**Almgren, 2012 [54]**	Sweden	Obesity, BMI, TG, TC, LDL, HDL, BG	BMI ≥ 35; 28 ≥ BMI ≤ 25; BMI < 25	424 children and 1522 nondiabetic adults, and 89 anonymous blood donors	7% in 1992–1998 to 15%–20% in 2002–2009, increase in obesity prevalence	SNA and ELISA
**Aldhoon-Hainerova, 2014 [55]**	Czech Republic	anthropometric (body weight, height, BMI, WC, fat mass), blood pressure, biochemical and hormonal (lipid profile, glucose, insulin, liver enzymes, adiponectin)	NA	1179 Czech adolescents (85 underweight, 506 normal weight, 160 overweight and 428 obese)	26.5% were positive for Adv36 antibodies (underweight: 22.3%; normal weight: 21.5%; overweight: 40.0% and obese: 28.0%)	ELISA
**Vander Wal, 2013 [61]**	USA	BMI, TC, HDL, LDL, TG	Mean BMI 33.77	73 youth aged 10–17 years	17 youth (23.3%; 2 boys, 15 girls) tested Ad-36 AB+ and 56 youth (76.6%; 14 boys, 42 girls) tested Ad-36 AB−.	SNA
**Lin, 2013 [100]**	MEXICO	Age, sex, Body FAT, BMI, Fasting glucose, Fasting insulin	Mean BMI 29.15	1,400 enrolled in the San Antonio Family Heart Study	Seropositive subjects (14.5%) had greater adiposity at baseline, compared with seronegative subjects.	SNA
**Laing, 2013 [101]**	USA	DXA	21 ≥ BMI ≤ 24	115 females aged 18 to 19 years	52% and 64% in normal-fat and high-fat groups	ELISA
**Vander Wal, 2013 [61]**	USA	TC, HDL, LDL, TG	Mean BMI 37.77	73 youth aged 10-17 years	17 youth (23.3%; 2 boys, 15 girls) tested Ad-36 AB+ and 56 youth (76.6%; 14 boys, 42 girls) tested Ad-36 AB–	SNA
**Parra-Rojas, 2013 [59]**	MEXICO	LDL, HDL, TG, Insulin, Fasting glucose, HOMA	NA	75 children with normal-weight and 82 with obesity	Seroprevalence was 73.9%. Ad-36 seropositivity had a higher prevalence in obese children than in normal weight group 58.6 *versus* 41.4%	ELISA
**Berger, 2014 [102]**	USA	TNF-α, IL-6, VEGF, MCP-1, DXA.	20 ≥ BMI ≤ 21	291 children aged 9-13 years (50% female, 49% black)	seropositivity [Ad36(+)] was 42%	ELISA
**Voss, 2014 [103]**	USA	NA	20–30 kg/m(2)	500 young, 18–22 years	seropositivity [Ad36(+)] was 20.8%	ELISA
**Karamese, 2015 [104]**	Turkey	TG, TC, LDL, TNF-α, IL-6, leptin	NA	146 children and 130 adults	27.1% and 6% in obese and non-obese children and 17.5% and 4% in obese and non-obese adults	ELISA
**Ergin, 2015 [105]**	Turkey	TC, TG, leptin	Obese BMI > 30; non-obese adults with BMI < 25	49 obese adults and 49 non-obese adults	seroprevalence was 12.2%, DNA was not detected	SNA, ELISA, PCR

Adv36 DNA has been isolated in few cases. Salehian *et al.* [48] described a patient with massive fat deposits in the thorax and abdomen arguing that the abnormal adipose tissue deposits might be caused by Adv36. Although this case seems in agreement with the increase of adipose tissue found in animals experimentally infected with Adv36, further studies are needed to understand the actual role of Adv36 in abnormal deposits and fat/lipomatosis in humans [48].

The association of natural Adv36 infection with obesity both in adults and children has been described (Table 1).

The first study of Ad36 infection in adult humans in the United States showed that about 30% of obese and 11% of non-obese had been infected, and there was a strong correlation of obesity with infection [49]. Other studies have confirmed these results (Table 1). The first study of Ad36 infection in adult humans in the United States showed that about 30% of obese and 11% of non-obese had been infected, and there was a strong correlation of obesity with infection [42]. Other studies have confirmed these results (Table 1), while few studies did not confirm this finding [50,51,52]. The average Adv36 prevalence ranged from 65% in Italy down to 6% in Belgium/Holland (Table 1). The Adv36 prevalence reported in obese children and non-obese children is 28% and 18%, respectively [53,54,55,56,57,58,59,60,61]. Two meta-analyses explored the association between Adv36 infection and obesity development. The first selected 10 studies. The authors found that Adv36 infection associates with the risk of obesity, but was not associated with abnormal metabolic markers including waist circumstance suggesting that the infection is more associated with accumulation of subcutaneous fat than that of visceral fat [62].

The second meta-analysis included 11 case-control studies, including 2508 obese subjects and 3005 control. The study identified an association between Ad36 infection and a significantly increased risk of obesity development, especially in children [63].

## 6. Molecular and Cellular Mechanisms Involved in Ad-36-Induced Glucose Uptake

Evidence emerging from animal and human studies suggests that some forms of obesity may actually lead to healthy obesity [24]. Several studies identified one Adv36 gene that mediates glucose disposal through the Ras/PI3K pathway to improve glucose uptake [64]. The glucose uptake increase may contribute to the improved glycemic control found in Adv36-infected animals [34].

*In vitro*, Adv36 induced adipogenic accumulation commitment, differentiation of adipocytes and increases the cellular glucose uptake [27,44].

**Figure 2:**
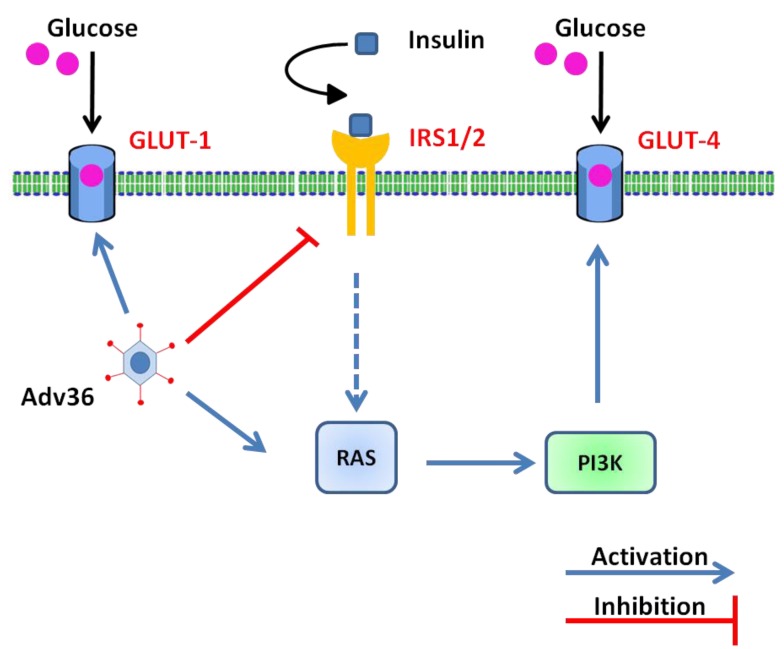
Adv36 mediates glucose uptake independently from insulin, adapted from [64]. Adv36 up-regulates the Phosphoinositide 3-kinase (PI3K) signaling via Ras, increasing cellular glucose uptake by glucose trasporters Glut1 and Glut4 despite a down-regulation of the Insulin Receptor Substrate (IRS) signaling.

In a work by Wang *et al.* [64], the authors showed that the infection with Adv36 increases glucose uptake, confirming previous data. *In vitro* experiments on Human Skeletal Muscle (HSKM) cells showed an increase of GLUT1 and GLUT4 gene expression mediated by Ras-activated PI 3-kinase pathway independent from insulin activation (Figure 2).

Considering the crucial role of skeletal muscle in glucose disposal, the ability to modulate its capacity to uptake glucose may have a major impact on systemic glycemic control. The property of Adv36 to improve skeletal muscle glucose uptake in an insulin-signaling independent manner is of great potential interest for the development of new drug targets in insulin resistance diabetes. Subsequently, many studies have focused on the virus E4orf1 gene that up-regulates the PI3K pathway (Figure 2) [42]. The E4orf1 gene is transcribed from the first open reading frame of the early gene of Adv36 and produces a 17 kDa protein (Figure 1) [42]. Adv36 increases cellular glucose uptake via Ras-mediated activation of the phosphatidyl inositol 3-kinase (PI3K), and improves hyperglycemia in mice, without reducing adiposity [65]. *In vitro* experiments showed that Adv36 significantly increases the absorption of glucose into 3T3-L1 preadipocytes and identified that the E4orf1 is “sufficient” to up-regulate the absorption of glucose [26]. The 3T3-L1 cell line expressing E4orf1 had an increased glucose uptake compared to null vector control cells. Moreover, E4orf1 up-regulated PI3K pathway and increased the Ras-molecule required to stimulates the Adv36-induced glucose uptake. Glucose uptake increases significantly in preadipocytes, adipocytes, or myoblasts derived from 3T3-L1 cells transiently transfected with E4orf1. Thus, the attractive anti-hyperglycemic effect of Adv36 could be mediated by the E4orf1 protein, which may offer a novel ligand to develop hypoglycemic drugs. Cellular uptake of glucose can be improved by up-regulation of Ras signaling in either insulin-dependent or insulin-independent diabetes. In the presence of an intact insulin signal, Ras plays a negligible role in glucose uptake. On the contrary, when the insulin signal is reduced like in obesity or diabetes, the insulin-independent Ras pathway can be useful to improve the glucose disposal. Adv36 increases cellular uptake of glucose by upregulating the Ras/Glut4pathway (Figure 2). These data improved the knowledge on the role of E4orf1 as a mediator of Adv36-induced glucose uptake [26].

The metabolic effects of E4orf1 are encouraging, and may provide new targets for signaling to prevent NAFLD and insulin resistance even in the presence of a high fat diet. There are limitations to these studies because all results are from *in vitro* experiments and should be verified *in vivo.* E4orf1 is not a secretory protein, and therefore does not have a cell surface receptor for cell entry. Thus, E4orf1 provides a valuable model to exogenously modulate hepatic glucose and lipid metabolism [66].

In summary, Adv36 may serve two different purposes, on the one hand, to explain the pathophysiology of certain cases of obesity and, on the other hand, to provide a potential weapon for improving insulin resistance, regardless of the consumption of fat.

Collectively, these results can provide a model to develop new agents for the treatment of hyperglycemia associated with obesity, and type 1 or type 2 diabetes [25]. Although the *in vivo* efficacy and safety of E4orf1 in improving hyperglycemia remain unknown, and an appropriate drug delivery system is required, Adv36 E4orf1 offers a research opportunity to develop new anti-diabetic agents with new conceptually advances for the use of a rather unconventional source, viral proteins, in the anti-diabetic drug development [25].

## 7. Adenovirus 36 and Immune Response

### 7.1. Inflammation: MCP-1 (Macrophage Chemoattractant Protein I) and Adv36

Obese subjects have altered the overall number of circulating T-cells and obesity has been associated with decreased thymic output of naive T-cells [67,68,69]. When analyzed by flow cytometry, decreased CD8 T-cell populations and increased or decreased numbers of CD4 T-cells compared with lean controls are found [70]. The inflammatory network in obesity may present different immunologic phenotypes depending on the degree of metabolic disorder. In lean individuals, adipose tissue is mainly characterized by anti-inflammatory condition, while obesity shifts towards a pro-inflammatory status. Obesity negatively impacts the ability of dendritic cells (DCs) to mature and elicit appropriate T-cell responses to a general stimulus. This may contribute to the increased susceptibility to viral infection observed in severe obesity [71]. Adv36 infection could induce obesity through inflammation, and MCP-1 may be a key regulator of adenovirus 36-induced obesity in Adv36-infected mice [72]. Thus, Adv36 could cause chronic inflammation by increasing the levels of MCP-1, activating nuclear factor κB (NF-κB), inducing the infiltration of macrophages into adipocytes, and altering lipid metabolism [72]. The correlation between obesity and inflammation has been known for decades from epidemiological and cellular studies [73,74]. Hypertrophic adipocytes may produce pro-inflammatory cytokines such as MCP-1, TNF-α, resistin, and plasminogen activator inhibitor-1 [75,76]. It was previously found that Adv36 infection triggers the inflammatory pathway in human mesenchymal stem cells, which was confirmed by microarray analysis [77].

It has been reported that MCP-1 is responsible for obesity, insulin resistance, and steatosis in MCP-1 transgenic mice and obese mice [78] and inhibition of MCP-1 ameliorates insulin resistance and hepatic steatosis [78]. MCP-1 plays a role in the recruitment of monocytes and macrophages into adipose tissue [78,79] and TNF-α increases the number of macrophages in adipose tissue [80,81]. Macrophages upregulate production of the anti-inflammatory cytokine IL-10 and downregulate synthesis of pro-inflammatory cytokines. Functionally, there are macrophages that are associated with the repair of injured tissues and the resolution of inflammation. So, it has been shown that the macrophages accumulate in the adipose tissues of obese mice mainly expressing genes associated with an M1 or “classically activated” macrophage phenotype, whereas adipose tissue macrophages from lean mice express genes associated with an M2 or “alternatively activated” macrophage phenotype [82]. Adv36 infection increased M1 macrophages migration into adipocytes by activating nuclear factor κB (NFkB), which induced the release of pro-inflammatory citokynes. In the study of Na *et al.* [77] on *in vivo* experimental infection in mice, Adv36 infection stimulates an inflammatory state due to the increased level of MCP-1 through the activation of NFkB, which in turn induces the infiltration of macrophages into adipocytes. Adv36 infection increases MCP-1, and MCP-1 might function to induce adipogenesis via MCP-1-related factors in adipocytes. Furthermore, increased MCP-1 triggers macrophage infiltration into adipocytes and inflammation may play a role in maintaining obesity [72]. Therefore, on the basis of these data, we could assume that there is a relationship between Adv36-induced obesity and inflammation.

### 7.2. Effect of Adv36 on Leptin

Conventionally, obesity can be considered an over accumulation of white adipose tissue (WAT). Although adipocytes occupy the bulk of the volume of WAT, adipose tissue also includes many more cells types, including a diverse population of preadipocytes, macrophages, endothelial cells, fibroblasts and leukocytes [83]. Adipose tissue, traditionally thought as a passive storage for triglycerides and energy, but in the past two decades it has been linked to the production of several hormones, pro-inflammatory chemokines, adipokines and cytokines, including leptin, adiponectin, resistin, visfatin, B-cell activating factor of the Tumor Necrosis Factor (TNF) family, TNF-like weak inducer of apoptosis, a proliferation inducing ligand, TNF-α, omentin and MCP-1 [5]. Based on this concept, adipose tissue has been defined an endocrine organ. In the obese state, secretion of these adipokines is altered in correlation to the increased adipose tissue mass [84,85]. To date, adipokine modulation of immune function by leptin is the best-characterized link between obesity and immune function. Leptin plays a role in many diverse physiological processes but is primarily involved in energy homeostasis and satiety [86,87].

Leptin acts as a general signal of energy reserves and modulates food intake. Leptin levels increase proportionately to adipose mass resulting in high circulating leptin concentrations in obese individuals [88,89].

**Figure 3:**
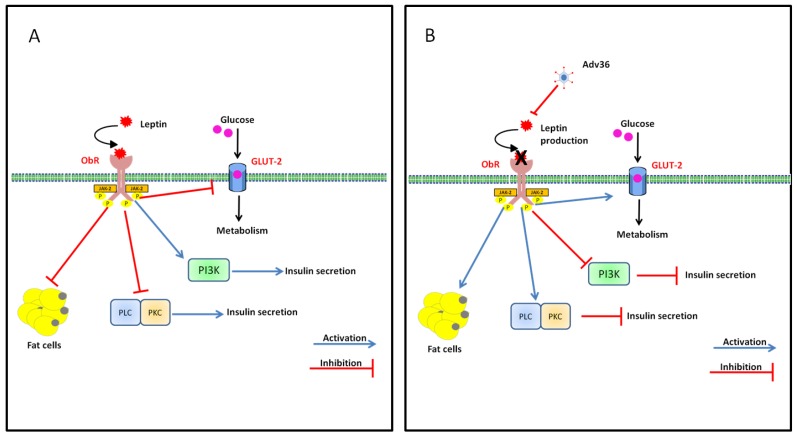
Adv36 and leptin. (**A**) Leptin binding to its receptor (ObRb) activates the associated JAK-2 tyrosine kinase. Leptin inhibits glucose transport through GLUT-2, and activates PI3K. Additionally, Phosphoinositide 3-kinase (PI3K) activation by leptin reduces Cyclic adenosine monophosphate (cAMP) levels and activates the Protein kinase A (PKA) pathway. Leptin can also inhibit the phospholipase C (PLC)/protein kinase C (PKC) pathway; (**B**) Adv36 inhibits leptin production. The results are decreased insulin release and increased lipid accumulation.

**Figure 4:**
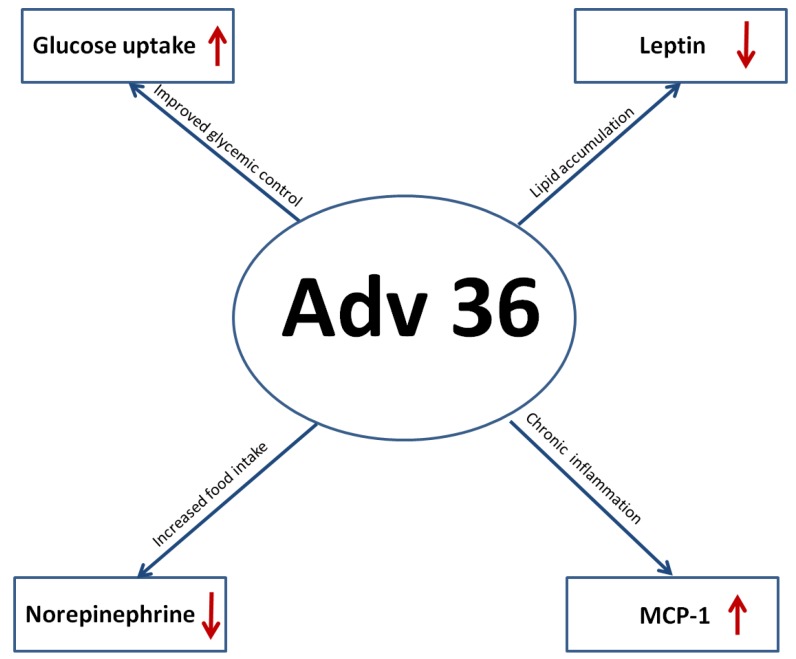
Proposed mechanisms underlying the effects of Adv36 in infected individuals.

The leptin receptor is expressed by B and T lymphocytes and may directly modulate the T and B responses [90]. Leptin seems to exert its effects on immune cells through the JAK /STAT pathway. In peripheral blood mononuclear cells, leptin increases JAK2/3 and STAT3 phosphorylation, which promote proliferation and activation of T lymphocytes upon mitogen stimulation. In terms of infectious disease, the general consensus seems to be that leptin has an anti-inflammatory role, while at the same time serving a protective capacity against infections [91]. Inflammation is used as a localized, protective response to infection and changes in body weight and metabolic state are often associated with acute or chronic inflammatory processes resulting from infection [38]. The inhibition of leptin gene expression by Adv36 infection may increase lipid accumulation and obesity prevalence reported that human cells infected with Adv36 showed greater differentiation and higher levels of lipid accumulation than non-infected control cells [43] (Figure 3). Adv36 infection may increase appetite and food intake by decreasing norepinephrine levels and leptin [92], thereby increasing obesity prevalence (Figure 4).

## 8. Proof-of-Concept of a Vaccine Using UV Inactivated Adv36

Adv36 infection is characterized by greater adiposity and inflammation. To investigate the possibility that a candidate prophylactic vaccine could protect Adv36-induced obesity and inflammation an inactivated Adv36 vaccine was tested [37]. Mice were inoculated with live Adv36, UV-inactivated Adv36, or with medium alone. Live Adv36 increased the size of the epididymal fat pad at four days post-inoculation, whereas UV-inactivated Adv36 prevented it. In another study, mice were immunized with purified and ultraviolet-irradiated virus as candidate vaccine, live Adv36 was injected into mice as a challenge test. Unvaccinated mice (control group) were immunized with phosphate-buffered saline and then challenged with live Adv36. The control group showed 17% greater body weight and 20% more epididymal fats compared with the vaccinated group. Moreover, the vaccinated group had decreased serum levels of pro-inflammatory cytokines, and infiltrated immune cells in fat tissue [93]. Therefore, the vaccine was able to protect against Adv36-increased body weight and fat as well as inflammatory states after challenge. These results could provide proof-of-concept for prophylactic vaccination against virus-induced adiposity [94].

## 9. Conclusions

Obesity is a multifactorial pathology and the understanding of the different contributing factors is crucial for its efficient management.

Recent studies have shown a possible correlation between obesity and Adv36 viral infections. Animals infected with Adv36 show increase of body weight and physiological changes, increased glucose absorption and decreased secretion of leptin and cholesterol (Figure 4).

The Adv36 infections should therefore be considered as a possible risk factor for obesity and this could be a potential new way to investigate on the worldwide epidemic of obesity. Further research on viruses contributing to obesity is essential. Studying the favorable effect of Adv36 on metabolic consequences of obesity may provide insight to novel treatments that improve glycemic control despite adiposity. Identifying the viral protein responsible for influencing glucose disposal may help in developing novel anti-diabetic therapeutic agents.
